# Synthesis, Characterization, and Interaction with Biomolecules of Platinum(II) Complexes with Shikimic Acid-Based Ligands

**DOI:** 10.1155/2013/565032

**Published:** 2013-03-05

**Authors:** Yan Peng, Min-Min Zhang, Zhen-Feng Chen, Kun Hu, Yan-Cheng Liu, Xia Chen, Hong Liang

**Affiliations:** State Key Laboratory Cultivation Base for the Chemistry and Molecular Engineering of Medicinal Resources, School of Chemistry and Chemical Engineering of Guangxi Normal University, Guilin 541004, China

## Abstract

Starting from the active ingredient shikimic acid (SA) of traditional Chinese medicine and NH_2_(CH_2_)_*n*_OH, (*n* = 2–6), we have synthesized a series of new water-soluble Pt(II) complexes PtL^a–e^Cl_2_, where L^a–e^ are chelating diamine ligands with carbon chain covalently attached to SA (L^a–e^ = SA-NH(CH_2_)_*n*_NHCH_2_CH_2_NH_2_; L^a^, *n* = 2; L^b^, *n* = 3; L^c^, *n* = 4; L^d^, *n* = 5; L^e^, *n* = 6). The results of the elemental analysis, LC-MS, capillary electrophoresis, and ^1^H, ^13^C NMR indicated that there was only one product (isomer) formed under the present experimental conditions, in which the coordinate mode of PtL^a–e^Cl_2_ was two-amine bidentate. Their *in vitro* cytotoxic activities were evaluated by MTT method, where these compounds only exhibited low cytotoxicity towards BEL7404, which should correlate their low lipophilicity. The interactions of the five Pt(II) complexes with DNA were investigated by agarose gel electrophoresis, which suggests that the Pt(II) complexes could induce DNA alteration. We also studied the interactions of the Pt(II) complexes with 5′-GMP with ESI-MS and ^1^H NMR and found that PtL^b^Cl_2_, PtL^c^Cl_2_, and PtL^d^Cl_2_ could react with 5′-GMP to form mono-GMP and bis-GMP adducts. Furthermore, the cell-cycle analysis revealed that PtL^b^Cl_2_, PtL^c^Cl_2_ cause cell G_2_-phase arrest after incubation for 72 h. Overall, these water-soluble Pt(II) complexes interact with DNA mainly through covalent binding, which blocks the DNA synthesis and replication and thus induces cytotoxicity that weakens as the length of carbon chain increases.

## 1. Introduction

As Pt(II) complexes have demonstrated successful clinical application of cisplatin for its anticancer effects, new platinum-based anticancer drugs are highly desired [1–12]. Up to now, there have been five platinum-based anticancer drugs used in clinical applications, including three FDA-approved platinum compounds: cisplatin, carboplatin, oxaliplatin, nedaplatin used in Japan, and lobaplatin approved for use in China. In addition, new and nontraditional compounds picoplatin (AMD473) [13] and ProLindac [14] as well as platinum(IV) complexes, such as satraplatin [15], tetraplatin, tetrachloro-trans-R, R-cyclohexane-1,2-diamine platinum (IV), and ipropltin (dichlorodihydroxobis(isopropylamine)platinum (IV) [16, 17], are being evaluated for clinical trials. Nevertheless, their effectiveness is still hindered by clinical problems, such as acquired or intrinsic resistance that limits the spectrum of cancers that can be treated, and high toxicity leading to side effects and limiting the dose that can be registered [18]. In the past three decades, substantial efforts have been directed to the tactics that can improve cellular accumulation, oral bioavailability, lifetime in blood, and tumor targeting [13].

 During the past two decades, water-soluble platinum(II) complexes have been synthesized, which cannot only retain antitumor activity but also be effectively absorbed after oral administration [19]. The most common approach to prepare these compounds is a structural approach, in which the chloride ligands are replaced by chelating carboxylates, oxalate, and glycolate [20–22]. Besides, there are many other methods, such as platinum terpyridine complexes with glycosylated acetylide and arylacetylide ligands [23], water-soluble macromolecular platinum conjugates [24, 25], water-soluble porphyrin-Pt(II) conjugates [26, 27], formation of water-soluble organometallic analogues of oxaliplatin [28], or supramolecular nanoencapsulation technique [29]. However, the water-soluble platinum complexes archived by means of amine ligands instead of ammonia are most intriguing because the carrier ligands provide broad spectrum of antitumor activity [30]. For instance, water-soluble platinum(II) complexes of diamine chelating ligands bearing amino-acid type substituents [31], the carbohydrate-metal complexes, have been proved a potential effective method [32–36]. But the synthesis of this complex is extremely laborious, which also lacks clinical data. By contrast, shikimic acid (3R,4S,5R-trihydroxy-1-cyclohexane-1-carboxylic acid, SA), an active ingredient isolated from traditional Chinese medicine *Illicium verum Hook. f*. grown in Guangxi province of China, has exhibited good water solubility. It is well used as DNA vaccine carrier [37] and [^99 m^Tc(CO)_3_]-labeled bombesin to reduce both hepatic uptake and renal retention [38–40]. Though Farrell and coauthors have reported shikimic acid complexes of platinum [41], diamine coupled shikimic acid complexes of platinum remain unstudied. In this work, we have synthesized a series of platinum complexes (PtL^a–e^Cl_2_) with diamine coupled shikimic acid ligands L^a–e^ (L^a–e^ = SA-NH(CH_2_)_*n*_NHCH_2_CH_2_NH_2_; L^a^, *n* = 2; L^b^, *n* = 3; L^c^, *n* = 4; L^d^, *n* = 5; L^e^, *n* = 6) and investigated their cytotoxicity and DNA-binding properties.

## 2. Materials and Methods

### 2.1. Materials

All chemicals were purchased from commercial sources and used as received. All solvents were of analytical grade and used without further purification unless otherwise specified. The synthesized Pt(II) complexes as well as the ligands were dissolved in H_2_O at a concentration of 5.0 mM as stock solutions to prepare the DNA binding studies. Disodium salt of guanosine-5′-monophosphate (5′-GMP) was purchased from Sigma. pUC19 plasmid DNA was purchased from Takara Biotech Co. Ltd., Dalian of China. Cancer cell lines were obtained from Shanghai Institutes for Biological Sciences of China.

### 2.2. Instrumentation and Methods

^1^H and ^13^C NMR spectra were recorded by a Bruker AV-500 NMR spectrometer with chemical shift (in ppm) relative to tetramethylsilane. Elemental analyses (C, H, N) were performed on a Perkin Elmer Series II CHNS/O 2400 analytical instrument. ESI mass spectra were measured on a Bruker HCT Electrospray Ionization Mass Spectrometer. The purity of the platinum(II) complexes was performed on Thermo Fisher Scientific Exactive LC-MS Spectrometer. Capillary electrophoresis was recorded on Agilent HP3D High Performance Capillary Electrophoresis. Flow-cytometric analysis was undertaken by using a FACScan fluorescence-activated cell sorter (FACS).

### 2.3. Synthesis

**Scheme 1:**
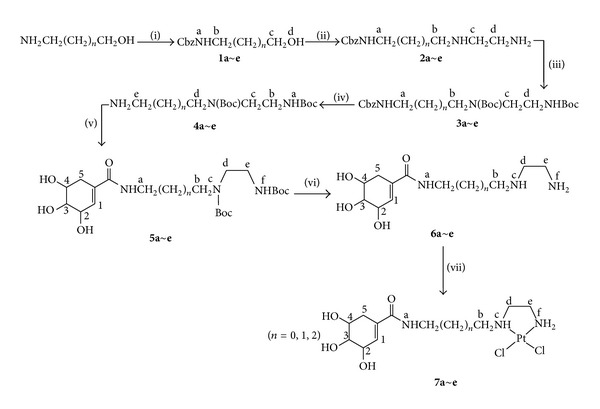
Synthesis route for the shikimic acid derivate ligands and Pt(II) complexes (*n* = 2, 3, 4, 5, 6): (i) CbzCl, NaHCO_3_, H_2_O; (ii) MsCl/py/20°C, excess NH_2_(CH_2_)_2_NH_2_; (iii) (t-BuO)_2_CO, CH_2_Cl_2_; (iv) HCO_2_NH_4_/Pd/C; (v) Shikimic acid, DCC, HOBt; (vi) conc. HCl/MeOH/CH_2_Cl_2_; (vii) Na_2_CO_3_, K_2_PtCl_4_.

The compounds **1a~e**, **2a~e**, and **3a~e** were prepared following the methods reported by Holmes et al. [42, 43]. The data are in good agreement with the literatures. The compounds **4a~e**, **5a~e**, **6a~e** (L^a–e^), and **7a~e** (PtL^a–e^Cl_2_) were synthesized via modified methods that were reported by Srinivas et al. [37–40, 44–47] (see Scheme 1).

*Compound* ***1a***.  ^1^H NMR (500 MHz, CDCl_3_): *δ* = 7.36 (m, 5H, C_6_H_5_), 5.39 (br s, 1H, H(a)), 5.11 (s, 2H, CH_2_), 3.69 (t,  *J* = 4.5 Hz, 2H, H(c)), 3.35 (m, 2H, H(b)); ESI-MS  *m*/*z*  217.84 [M + Na]^+^.

*Compound* ***1b***.  ^1^H NMR (500 MHz, CDCl_3_): *δ* = 7.36 (m, 5H, C_6_H_5_), 5.47 (br s, 1H, H(a)), 5.12 (s, 2H, CH_2_), 3.66 (t, *J* = 5.5 Hz, 2H, H(c)), 3.32 (m, 2H, H(b)), 1.70 (m, 4H, 2 × CH_2_); ESI-MS  *m*/*z*  209.85 [M + H]^+^.

*Compound* ***1c***. ^1^H NMR (500 MHz, CDCl_3_): *δ* = 7.39 (m, 5H, C_6_H_5_), 5.20 (br s, 1H, H(a)), 5.12 (s, 2H, CH_2_), 3.67 (m, 2H, H(c)), 3.25 (m, 2H, H(b)), 1.91 (br s, 1H, H(d)), 1.61 (m, 4H, 2 × CH_2_); ESI-MS  *m*/*z*  223.85 [M + H]^+^.

*Compound* ***1d***. ^1^H NMR (500 MHz, CDCl_3_): *δ* = 7.38 (m, 5H, C_6_H_5_), 5.12 (s, 2H, CH_2_), 3.66 (t, *J* = 6.5 Hz, 2H, H(c)), 3.23 (m, 2H, H(b)), 1.57 (m, 4H, 2 × CH_2_), 1.42 (m, 2H, CH_2_); ESI-MS  *m*/*z*  259.92 [M + Na]^+^.

*Compound* ***1e***. ^1^H NMR (500 MHz, CDCl_3_): *δ* = 7.35 (m, 5H, C_6_H_5_), 5.09 (s, 2H, CH_2_), 3.62 (t, *J* = 6.5 Hz, 2H, H(c)), 3.20 (m, 2H, H(b)), 1.66 (br s, 1H, H(d)), 1.54 (m, 4H, 2 × CH_2_), 1.36 (m, 4H, 2 × CH_2_); ESI-MS  *m*/*z*  274.01 [M + Na]^+^.

*Compound* ***2a***. ^1^H NMR (500 MHz, CDCl_3_): *δ* = 7.34 (m, 5H, C_6_H_5_), 5.02 (s, 2H, CH_2_), 3.24 (m, 2H, H(a)), 2.90 (m, 4H, H(b), and H(c)), 2.69 (m, 2H, H(d)); ESI-MS  *m*/*z*  238.10 [M + H]^+^.

*Compound* ***2b***.  ^1^H NMR (500 MHz, CDCl_3_): *δ* = 7.30 (m, 5H, C_6_H_5_), 5.04 (s, 2H, CH_2_), 3.45 (m, 2H, H(a)), 3.30 (m, 4H, H(b), and H(c)), 2.89 (m, 2H, H(d)), 1.89 (m, 2H, CH_2_); ESI-MS  *m*/*z*  251.95 [M + H]^+^.

*Compound* ***2c***. ^1^H NMR (500 MHz, CDCl_3_): *δ* = 7.34 (m, 5H, C_6_H_5_), 5.09 (s, 2H, CH_2_), 3.27 (t, *J* = 6.0 Hz, 2H, H(a)), 2.96 (m, 2H, H(c)), 2.86 (m, 4H, H(b), and H(d)), 1.90 (m, 4H, 2 × CH_2_); ESI-MS  *m*/*z*  266.91 [M + H]^+^.

*Compound* ***2d***. ^1^H NMR (500 MHz, CDCl_3_): *δ* = 7.37 (m, 5H, C_6_H_5_), 5.10 (s, 2H, CH_2_), 3.20 (m, 2H, H(a)), 2.82 (t, *J* = 6.0 Hz, 2H, H(c)), 2.67 (m, 2H, H(b)), 2.41 (m, 2H, H(d)), 1.53 (m, 4H, 2 × CH_2_), 1.36 (m, 2H, CH_2_); ESI-MS  *m*/*z*  280.01 [M+H]^+^.

*Compound* ***2e***. ^1^H NMR (500 MHz, CDCl_3_): *δ* = 7.34 (m, 5H, C_6_H_5_), 5.09 (s, 2H, CH_2_), 3.16 (m, 2H, H(a)), 2.40 (m, 6H, H(b), H(c), and H(d)), 1.50 (m, 4H, 2 × CH_2_), 1.33 (m, 4H, 2 × CH_2_); ESI-MS  *m*/*z*  294.06 [M + H]^+^.

*Compound* ***3a***. ^1^H NMR (500 MHz, CDCl_3_): *δ* = 7.36 (m, 5H, C_6_H_5_), 5.11 (s, 2H, CH_2_), 3.33 (m, 8H, H(a), H(b), H(c), and H(d)), 1.47 (s, 9H, 3 × CH_3_), 1.44 (s, 9H, 3 × CH_3_); ESI-MS  *m*/*z*  460.23 [M + Na]^+^.

*Compound* ***3b***. ^1^H NMR (500 MHz, CDCl_3_): *δ* = 7.35 (m, 5H, C_6_H_5_), 5.09 (s, 2H, CH_2_), 3.25 (m, 8H, H(a), H(b), H(c), and H(d)), 1.46 (m, 11H, CH_2_ and 3 × CH_3_), 1.43 (s, 9H, 3 × CH_3_); ESI-MS  *m*/*z*  486.21, 488.16 [M + Cl]^−^.

*Compound* ***3c***. ^1^H NMR (500 MHz, CDCl_3_): *δ* = 7.36 (m, 5H, C_6_H_5_), 5.11 (s, 2H, CH_2_), 3.23 (m, 8H, H(a), H(b), H(c), and H(d)), 1.55 (m, 4H, 2 × CH_2_), 1.47 (s, 9H, 3 × CH_3_), 1.44 (s, 9H, 3 × CH_3_); ESI-MS  *m*/*z*  500.21, 502.18 [M + Cl]^−^.

*Compound* ***3d***. ^1^H NMR (500 MHz, CDCl_3_): *δ* = 7.36 (m, 5H, C_6_H_5_), 5.08 (s, 2H, CH_2_), 3.26 (m, 8H, H(a), H(b), H(c), and H(d)), 1.51 (m, 4H, 2 × CH_2_), 1.44 (s, 9H, 3 × CH_3_), 1.42 (s, 9H, 3 × CH_3_), 1.28 (m, 2H, CH_2_); ESI-MS  *m*/*z*  514.22, 516.17 [M + Cl]^−^.

*Compound* ***3e***. ^1^H NMR (500 MHz, CDCl_3_): *δ* = 7.34 (m, 5H, C_6_H_5_), 5.08 (s, 2H, CH_2_), 3.27 (m, 8H, H(a), H(b), H(c) and H(d)), 1.49 (m, 4H, 2 × CH_2_), 1.45 (s, 9H, 3 × CH_3_), 1.42 (s, 9H, 3 × CH_3_), 1.27 (m, 4H, 2 × CH_2_); ESI-MS  *m*/*z*  528.24, 530.21 [M + Cl]^−^.

*Compound* ***4a***. The synthesis and characterization of **4a** have been well established [42], but in this work, the synthetic routine was modified [44–47]. 10% Pd/C (0.4 g) and ammonium formate (5.04 g, 80.0 mmol) suspension in 30 mL MeOH were added to a solution of **3a** (4.37 g, 10.0 mmol) in MeOH (50 mL) under nitrogen. The reaction mixture was vigorously stirred for 2.5 h at 40°C. This solution was allowed to cool and filtered through Celite then evaporated under reduced pressure. The residual ammonium formate was removed by repeated evaporation with CH_2_Cl_2_. The resulting oil was obtained. Yield: 98%. ^1^H NMR (500 MHz, CDCl_3_): *δ* = 8.45 (br s, 1H, H(a)), 3.52 (t, *J* = 6.0 Hz, 2H, H(b)), 3.34 (m, 2H, H(c)), 3.25 (m, 2H, H(d)), 3.11 (m, 2H, H(e)), 1.46 (s, 9H, 3 × CH_3_), 1.42 (s, 9H, 3 × CH_3_); ^13^C NMR (125 MHz, CDCl_3_): *δ* = 167.8, 155.8 CO, 47.3, 46.1 NCH_2_CH_2_NH, 38.9, 38.1 NH_2_CH_2_CH_2_N, 80.4, 78.8 C(CH_3_)_3_, 27.9 CH_3_; ESI-MS:  *m*/*z*: 304.10 [M + H]^+^.

*Compound* ***4b***. The procedure was similar to that for **4a** except that **3b** was used. Yield: 95%. ^1^H NMR (500 MHz, CDCl_3_): *δ* = 8.37 (br s, 1H, H(a)), 3.19 (m, 4H, H(b), and H(c)), 3.12 (t, *J* = 6.5 Hz, 2H, H(d)), 2.82 (m, 2H, H(e)), 1.81 (m, 2H, CH_2_), 1.34 (s, 9H, 3 × CH_3_), 1.31 (s, 9H, 3 × CH_3_); ^13^C NMR (125 MHz, CDCl_3_): *δ* = 168.2, 155.6 CO, 46.3, 43.6 NCH_2_CH_2_NH, 38.7, 36.3, 25.6 NH_2_CH_2_CH_2_CH_2_N, 79.9, 78.5 C(CH_3_)_3_, 27.8 6 × CH_3_; ESI-MS:  *m*/*z*: 318.16 [M + H]^+^.

*Compound* ***4c***. The procedure was similar to that for **4a** except that **3c** was used. Yield: 98%. ^1^H NMR (500 MHz, CDCl_3_): *δ* = 8.34 (br s, 1H, H(a)), 3.22 (m, 4H, H(b), and H(c)), 3.13 (m, 2H, H(d)), 2.67 (m, 2H, H(e)), 1.41 (m, 4H, 2 × CH_2_), 1.29 (s, 9H, 3 × CH_3_), 1.26 (s, 9H, 3 × CH_3_); ^13^C NMR (125 MHz, CDCl_3_) *δ* = 168.6, 155.5 CO, 46.6, 46.0 NCH_2_CH_2_NH, 39.5, 38.8, 27.1, 24.7 NH_2_CH_2_CH_2_CH_2_CH_2_N, 79.9, 78.5 C(CH_3_)_3_, 27.7 6 × CH_3_; ESI-MS:  *m*/*z*: 332.17 [M + H]^+^.

*Compound* ***4d***. The procedure was similar to that for **4a** except that **3d** was used. Yield: 94%. ^1^H NMR (500 MHz, CDCl_3_): *δ* = 8.37 (br s, 1H, H(a)), 3.15 (m, 4H, H(b), and H(c)), 3.09 (m, 2H, H(d)), 2.68 (m, 2H, H(e)), 1.39 (m, 4H, 2 × CH_2_), 1.31 (s, 9H, 3 × CH_3_), 1.28 (s, 9H, 3 × CH_3_), 1.18 (m, 2H, CH_2_); ^13^C NMR (125 MHz, CDCl_3_) *δ* = 168.7, 155.5 CO, 46.7, 45.9 NCH_2_CH_2_NH, 39.5, 38.8, 27.0, 23.1, 22.3 NH_2_CH_2_CH_2_CH_2_CH_2_CH_2_N, 79.0, 78.3 C(CH_3_)_3_, 27.7, 6 × CH_3_; ESI-MS:  *m*/*z*: 346.21 [M + H]^+^.

*Compound* ***4e***. The procedure was similar to that for **4a** except that **3e** was used. Yield: 96%. ^1^H NMR (500 MHz, CDCl_3_): *δ* = 8.32 (br s, 1H, H(a)), 3.06 (m, 6H, H(b), H(c), and H(d)), 2.60 (t, *J* = 7.0 Hz, 2H, H(b)), 1.39 (m, 4H, 2 × CH_2_), 1.28 (s, 9H, 3 × CH_3_), 1.25 (s, 9H, 3 × CH_3_), 1.19 (m, 2H, CH_2_), 1.12 (m, 2H, CH_2_); ^13^C NMR (125 MHz, CDCl_3_) *δ* = 168.6, 155.4 CO, 46.9, 45.8 NCH_2_CH_2_NH, 40.1, 38.9, 29.9, 27.3, 25.7 NH_2_CH_2_CH_2_CH_2_CH_2_CH_2_CH_2_N, 78.9, 78.3 C(CH_3_)_3_, 27.7 6 × CH_3_; ESI-MS:  *m*/*z*: 360.19 [M + H]^+^.

*Compound* ***5a***. This compound was synthesized by the modified method reported by Bowen et al. [48]. A solution of SA (1.65 g, 9.50 mmol), DCC (2.17 g, 10.5 mmol), and HOBT (1.42 g, 10.5 mmol) in DMF (20 mL) was stirred at 0°C for 1 h. Compound **4a** (0.288 g, 9.50 mmol) in CH_2_Cl_2_ (10 mL) was then added. After being stirred at room temperature for 24 h, the mixture was filtered and evaporated under reduced pressure to give the crude product that was purified by chromatography on silica gel using CHCl_3_/MeOH 20 : 1 as the eluent to produce the desired product **5a**. Yield: 58%. ^1^H NMR (500 MHz, CDCl_3_): *δ* = 6.41 (br s, 1H, H(1)), 4.35 (m, 1H, H(2)), 3.96 (m, 1H, H(3)), 3.60 (m, 1H, H(4)), 3.37 (m, 4H, H(d), and H(e)), 3.30 (m, 2H, H(a)), 3.22 (m, 2H, H(b)), 2.75 (m, 1H, H(5)), 2.18 (m, 1H, H(5)), 1.43 (s, 18H, 6 × CH_3_); ^13^C NMR (125 MHz, CDCl_3_): *δ* = 170.4, 155.9, 155.6 CO, 133.2, 130.3 C=CH, 72.2, 66.2, 65.7 CH–OH, 31.5 CH(OH)CH_2_, 45.5 NCH_2_CH_2_NH, 38.9, 38.3 NHCH_2_CH_2_N, 80.0, 78.7 C(CH_3_)_3_, 27.7 CH_3_; ESI-MS:  *m*/*z*: 494.26, 496.20 [M + Cl]^−^.

*Compound* ***5b***. The procedure was similar to that for **5a** except that **4b** was used. Yield: 60%. ^1^H NMR (500 MHz, CDCl_3_): *δ* = 6.34 (br s, 1H, H(1)), 4.28 (m, 1H, H(2)), 3.90 (m, 1H, H(3)), 3.54 (m, 1H, H(4)), 3.14 (m, 8H, H(a), H(b), H(d), and H(e)), 2.67 (m, 1H, H(5)), 2.14 (m, 1H, H(5)), 1.62 (m, 2H, CH_2_), 1.35 (s, 18H, 6 × CH_3_); ^13^C NMR (125 MHz, CDCl_3_) *δ* = 167.8, 155.6 CO, 133.4, 130.1 C=CH, 72.1, 66.2, 65.8 CH–OH, 31.5 CH(OH)CH_2_, 46.1, 44.0 NCH_2_CH_2_NH, 38.9, 36.2, 31.5 NHCH_2_CH_2_CH_2_N, 79.7, 78.6 C(CH_3_)_3_, 27.9 6 × CH_3_; ESI-MS:  *m*/*z*: 474.19 [M + H]^+^, 508.21, 510.21 [M + Cl]^−^.

*Compound* ***5c***. The procedure was similar to that for **5a** except that **4c** was used. Yield: 62%. ^1^H NMR (500 MHz, CDCl_3_): *δ* = 6.26 (br s, 1H, H(1)), 4.86 (m, 1H, H(2)), 3.86 (m, 1H, H(3)), 3.50 (m, 1H, H(4)), 3.10 (m, 8H, H(a), H(b), H(d), and H(e)), 2.63 (m, 1H, H(5)), 2.07 (m, 1H, H(5)), 1.39 (m, 4H, 2 × CH_2_), 1.29 (s, 18H, 6 × CH_3_); ^13^C NMR (125 MHz, CDCl_3_) *δ* = 168.0, 155.5, 155.0 CO, 133.4, 129.9 C=CH, 72.1, 66.2, 65.8 CH–OH, 31.6 CH(OH)CH_2_, 46.4, 46.1 NCH_2_CH_2_NH, 38.9, 36.2, 31.5 NHCH_2_CH_2_CH_2_CH_2_N, 79.2, 78.5 C(CH_3_)_3_, 27.9 6 × CH_3_; ESI-MS:  *m*/*z*: 488.16 [M + H]^+^, 522.21, 524.24 [M + Cl]^−^.

*Compound* ***5d***. The procedure was similar to that for **5a** except that **4d** was used. Yield: 61%. ^1^H NMR (500 MHz, CDCl_3_): *δ* = 6.26 (br s, 1H, H(1)), 4.23 (m, 1H, H(2)), 3.85 (m, 1H, H(3)), 3.49 (m, 1H, H(4)), 3.10 (m, 8H, H(a), H(b), H(d), and H(e)), 2.63 (m, 1H, H(5)), 2.05 (m, 1H, H(5)), 1.39 (m, 4H, 2 × CH_2_), 1.29 (s, 18H, 6 × CH_3_) 1.14 (m, 2H, CH_2_); ^13^C NMR (125 MHz, CDCl_3_) *δ* = 168.0, 155.5, 155.0 CO, 133.4, 129.9 C=CH, 72.1, 66.2, 65.8 CH–OH, 31.6 CH(OH)CH_2_, 46.7, 46.0 NCH_2_CH_2_NH, 39.1, 29.0, 28.5, 27.2, 23.6 NHCH_2_CH_2_CH_2_CH_2_CH_2_N, 79.1, 78.4 C(CH_3_)_3_, 27.8 6 × CH_3_; ESI-MS:  *m*/*z*: 502.21 [M + H]^+^, 536.23, 538.20 [M + Cl]^−^.

*Compound* ***5e***. The procedure was similar to that for **5a** except that **4e** was used. Yield: 61%. ^1^H NMR (500 MHz, CDCl_3_): *δ* = 6.29 (br s, 1H, H(1)), 4.26 (m, 1H, H(2)), 3.89 (m, 1H, H(3)), 3.52 (m, 1H, H(4)), 3.14 (m, 8H, H(a), H(b), H(d), and H(e)), 2.67 (m, 1H, H(5)), 2.10 (m, 1H, H(5)), 1.42 (m, 4H, 2 × CH_2_), 1.33 (s, 18H, 6 × CH_3_), 1.22 (m, 4H, 2 × CH_2_); ^13^C NMR (125 MHz, CDCl_3_) *δ* = 167.8, 155.6, 155.0 CO, 133.7, 129.7 C=CH, 72.3, 66.2, 65.8 CH–OH, 31.7 CH(OH)CH_2_, 46.7, 46.0 NCH_2_CH_2_NH, 39.2, 29.0, 28.7, 27.4, 26.1, 25.9 NHCH_2_CH_2_CH_2_CH_2_CH_2_CH_2_N, 79.1, 78.4 C(CH_3_)_3_, 27.8, 6 × CH_3_; ESI-MS:  *m*/*z*: 516.21 [M + H]^+^, 550.25, 552.24 [M + Cl]^−^.

*Compound* ***6a*** *(*L*^*a*^).* Compound **5a** (0.688 g, 1.50 mmol) was dissolved in CH_2_Cl_2_/EtOH (8 : 1, 25 mL), and 6 mol/L HCl (3 mL) was added with vigorously stirring for 20 min. Then, the solution was evaporated under reduced pressure to give a colourless oily solid. This was dissolved in distilled water (30 mL) and washed with CH_2_Cl_2_ (3 × 30 mL), from which the aqueous layer was concentrated to produce the desired compound **6a**. Yield: 83%. ^1^H NMR (500 MHz, CD_3_SOCD_3_): *δ* = 9.38 (br s, 2H, H(a), and 2-OH), 8.31 (br s, 3H, H(c), H(f), and 3-OH), 8.04 (br s, 1H, 4-OH), 6.33 (br s, 1H, H(1)), 4.12 (m, 1H, H(2)), 3.76 (m, 1H, H(3)), 3.44 (m, 3H, H(4), and CH_2_), 3.14 (m, 4H, H(d), and H(e)), 2.99 (t, *J* = 5.5 Hz, 2H, H(b)), 2.50 (m, 1H, H(5)), 1.98 (dd, *J* = 17.5 Hz, *J* = 4.0 Hz, 1H, H(5)); ^13^C NMR (125 MHz, D_2_O): *δ* = 170.5, CO, 132.2, 132.0, C=CH, 71.2, 66.4, 65.7 CH–OH, 30.8 CH(OH)CH_2_, 47.8, 44.3 NHCH_2_CH_2_NH_2_, 36.0, 35.5 NHCH_2_CH_2_NH; ESI-MS:  *m*/*z*: 260.02 [M + H]^+^, 294.02, 296.00 [M + Cl]^−^.

*Compound* ***6b*** *(*L*^*b*^).* The procedure was similar to that for **6a** except that **5b** was used. Yield: 84%. ^1^H NMR (500 MHz, CD_3_SOCD_3_): *δ* = 9.40 (br s, 2H, H(a), and 2-OH), 8.38 (br s, 3H, H(c), H(f), and 3-OH), 8.07 (br s, 1H, 4-OH), 6.32 (br s, 1H, H(1)), 4.17 (m, 1H, H(2)), 3.82 (m, 1H, H(3)), 3.50 (m, 1H, H(4)), 3.18 (m, 6H, CH_2_, H(d), and H(e)), 2.91 (m, 2H, CH_2_), 2.80 (m, 2H, H(b)), 2.46 (m, 1H, H(5)), 2.00 (dd, *J* = 17.5 Hz, *J* = 4.0 Hz, 1H, H(5)); ^13^C NMR (125 MHz, CD_3_SOCD_3_): *δ* = 170.0 CO, 132.5, 131.3 C=CH, 71.2, 66.3, 65.6 CH–OH, 30.9 CH(OH)CH_2_, 45.6, 44.1 NHCH_2_CH_2_NH_2_, 36.1, 35.5, 25.4 CH_2_CH_2_CH_2_; ESI-MS  *m*/*z*: 274.01 [M + H]^+^, 308.12, 310.00 [M + Cl]^−^.

*Compound* ***6c*** *(*L*^*c*^). *The procedure was similar to that for **6a** except that **5c** was used. Yield: 88%. ^1^H NMR (500 MHz, CD_3_SOCD_3_): *δ* = 9.19 (br s, 2H, H(a), and 2-OH), 8.23 (br s, 3H, H(c), H(f), and 3-OH), 7.85 (br s, 1H, 4-OH), 6.22 (br s, 1H, H(1)), 4.11 (m, 1H, H(2)), 3.75 (m, 1H, H(3)), 3.42 (m, 3H, H(4)), 3.09 (m, 4H, H(d), and H(e)), 3.04 (m, 2H, CH_2_), 2.86 (m, 2H, H(b)), 2.39 (m, 1H, H(5)), 1.93 (dd, *J* = 17.5 Hz, *J* = 4.5 Hz, 1H, H(5)), 1.53 (m, 2H, CH_2_), 1.42 (m, 2H, CH_2_); ^13^C NMR (125 MHz, CD_3_SOCD_3_): *δ* = 170.0 CO, 132.8, 130.6 C=CH, 71.0, 66.1, 65.5 CH–OH, 30.6 CH(OH)CH_2_, 47.5, 43.7 NHCH_2_CH_2_NH_2_, 38.4, 35.2, 25.1, 22.6 CH_2_CH_2_CH_2_CH_2_; ESI-MS  *m*/*z*: 288.02 [M + H]^+^, 322.12, 324.07 [M + Cl]^−^.

*Compound* ***6d*** *(*L*^*d*^).* The procedure was similar to that for **6a** except that **5d** was used. Yield: 86%. ^1^H NMR (500 MHz, CD_3_SOCD_3_): *δ* = 9.22 (br s, 2H, H(a), and 2-OH), 8.28 (br s, 3H, H(c), H(f), and 3-OH), 7.85 (br s, 1H, 4-OH), 6.27 (br s, 1H, H(1)), 4.17 (m, 1H, H(2)), 3.82 (m, 1H, H(3)), 3.50 (m, 1H, H(4)), 3.15 (m, 4H, H(d), and H(e)), 3.10 (m, 2H, CH_2_), 2.91 (m, 2H, H(b)), 2.45 (m, 1H, H(5)), 1.96 (dd,  *J* = 17.5 Hz,  *J* = 5.5 Hz, 1H, H(5)), 1.62 (m, 2H, H(b)), 1.44 (m, 2H, CH_2_), 1.30 (m, 2H, CH_2_); ^13^C NMR (125 MHz, CD_3_SOCD_3_): *δ* = 167.8 CO, 132.6, 132.1 C=CH, 71.3, 67.1, 66.0 CH–OH, 30.9 CH(OH)CH_2_, 47.4, 44.5 NHCH_2_CH_2_NH_2_, 39.0, 35.8, 28.9, 25.6, 23.7 CH_2_CH_2_CH_2_CH_2_CH_2_; ESI-MS  *m*/*z*: 302.05 [M + H]^+^, 336.09, 338.06 [M + Cl]^−^.

*Compound* ***6e*** *(*L*^*e*^).* The procedure was similar to that for **6a** except that **5e** was used. Yield: 85%. ^1^H NMR (500 MHz, CD_3_SOCD_3_): *δ* = 9.54 (br s, 2H, H(a), and 2-OH), 8.50 (br s, 3H, H(c), H(f), and 3-OH), 7.80 (br s, 1H, 4-OH), 6.28 (br s, 1H, H(1)), 4.18 (m, 1H, H(2)), 3.83 (m, 1H, H(3)), 3.50 (m, 1H, H(4)), 3.20 (m, 4H, H(d), and H(e)), 3.10 (m, 2H, CH_2_), 2.88 (m, 2H, H(b)), 2.48 (m, 1H, H(5)), 2.00 (dd, *J* = 17.5 Hz, *J* = 5.0 Hz, 1H, H(5)), 1.66 (m, 2H, CH_2_), 1.45 (m, 2H, CH_2_), 1.34 (m, 2H, CH_2_), 1.27 (m, 2H, CH_2_); ^13^C NMR (125 MHz, CD_3_SOCD_3_) *δ* = 167.0 CO, 131.9, 131.7 C=CH, 71.0, 66.7, 65.6 CH–OH, 30.6 CH(OH)CH_2_, 46.8, 44.1 NHCH_2_CH_2_NH_2_, 38.6, 35.3, 28.8, 25.9, 25.6, 25.4 CH_2_CH_2_CH_2_CH_2_CH_2_CH_2_. ESI-MS  *m*/*z*: 316.01 [M + H]^+^.

*Compound* ***7a*** *(*PtL*^*a*^*Cl*_2_).* Compound **6a** (L^a^) (0.415 g, 1.25 mmol) was dissolved in EtOH (5 mL) and water (2 mL), and the pH was adjusted to 8-9 with 0.25 M aqueous Na_2_CO_3_. A solution of K_2_PtCl_4_ (0.518 g, 1.25 mmol) in water (3 mL) was added dropwise, and the resulting mixture was stirred for 12 h in the dark at room temperature. The solvent was then removed and the residue purified by silica gel chromatography using MeOH as the eluent to afford the product as a yellow solid. Yield: 78%. Elemental analysis (%) calcd. for C_11_H_21_Cl_2_N_3_O_4_Pt·H_2_O: C 24.32, H 4.27, N 7.73; found C 24.11, H 4.20, N 7.78; ^1^H NMR (500 MHz, CD_3_SOCD_3_): *δ* = 8.06 (m, 1H, H(a)), 7.02 (br s, 1H, H(c)), 6.32 (br s, 2H, H(f)), 6.39 (br s, 1H, H(1)), 4.82 (br s, 1H, 2-OH), 4.57 (d, *J* = 6.5 Hz, 1H, 3-OH), 4.74 (br s, 1H, 4-OH), 4.14 (m, 1H, H(2)), 3.77 (m, 1H, H(3)), 3.45 (m, 1H, H(4)), 3.11 (m, 2H, CH_2_), 2.87 (m, 2H, H(b), Pt satellites are observed as shoulders), 2.62 (m, 4H, H(d), and H(e), Pt satellites are observed as shoulders), 1.98 (m, 1H, H(5)), 2.44 (m, 1H, H(5)); ^13^C NMR (125 MHz, D_2_O): *δ* = 170.3 CO, 132.9, 131.8 C=CH, 71.6, 66.6, 66.0 CH–OH, 31.1 CH(OH)CH_2_, 55.7, 51.5 NHCH_2_CH_2_NH_2_, 46.7, 36.8 NHCH_2_CH_2_NH; ESI-MS:  *m*/*z*: 565.95, 566.98, 567.96, 568.97, 570.00 [M-Cl + DMSO]^+^.

*Compound* ***7b*** *(*PtL*^*b*^*Cl*_2_).* The procedure was similar to that for **7a** except that **6b** (L^b^) was used. Yield: 82%. Elemental analysis (%) calcd. for C_12_H_23_Cl_2_N_3_O_4_Pt·2H_2_O: C 25.05, H 4.73, N 7.30; found C 25.12, H 4.64, N 7.39; ^1^H NMR (500 MHz, CD_3_SOCD_3_): *δ* = 7.94 (m, 1H, H(a)), 6.96 (br s, 1H, H(c)), 6.29 (br s, 2H, H(f)), 6.24 (br s, 1H, H(1)), 4.83 (d, *J* = 3.0 Hz, 1H, 2-OH), 4.73 (d, *J* = 7.0 Hz, 1H, 3-OH), 4.57 (d, 1H, *J* = 4.5 Hz, 4-OH), 4.18 (m, 1H, H(2)), 3.82 (m, 1H, H(3)), 3.51 (m, 1H, H(4)), 3.15 (t, *J* = 6.0 Hz, 2H, CH_2_), 2.84 (m, 2H, H(b), Pt satellites are observed as shoulders), 2.58 (m, 4H, H(d) and H(e), Pt satellites are observed as shoulders), 2.47 (m, 1H, H(5)), 1.98 (m, 1H, H(5)), 1.75 (m, 2H, CH_2_); ^13^C NMR (125 MHz, D_2_O): *δ* = 170.5 CO, 133.4, 131.1 C=CH, 71.7, 66.6, 66.0 CH–OH, 31.3 CH(OH)CH_2_, 55.7, 50.5 NHCH_2_CH_2_NH_2_, 46.9, 36.9, 26.8 CH_2_CH_2_CH_2_; ESI-MS:  *m*/*z*: 572.08, 573.16, 574.09, 575.09, 576.07, 577.01, 578.09 [M + Cl]^−^.

*Compound* ***7c*** *(*PtL*^*c*^*Cl*_2_).* The procedure was similar to that for **7a** except that **6c** (L^c^) was used. Yield: 85%. Elemental analysis (%) calcd. for C_13_H_25_Cl_2_N_3_O_4_Pt·2H_2_O: C 26.49, H 4.96, N 7.13; found C 26.63, H 4.87, N 7.24; ^1^H NMR (500 MHz, CD_3_SOCD_3_): *δ* = 7.91 (br s, 1H, H(a)), 6.99 (br s, 1H, H(c)), 6.43 (br s, 2H, H(f)), 6.29 (br s, 1H, H(1)), 4.88 (d, *J* = 4.0 Hz, 1H, 2-OH), 4.79 (d, *J* = 6.5 Hz, 1H, 3-OH), 4.63 (d, *J* = 5.0 Hz, 1H, 4-OH), 4.18 (m, 1H, H(2)), 3.82 (m, 1H, H(3)), 3.51 (m, 1H, H(4)), 3.11 (t, *J* = 5.0 Hz, 2H, CH_2_), 2.84 (m, 2H, H(b), Pt satellites are observed as shoulders), 2.59 (m, 4H, H(d), and H(e), Pt satellites are observed as shoulders), 2.47 (m, 1H, H(5)), 1.97 (dd,  *J* = 17.5 Hz, *J* = 3.5 Hz, 1H, H(5)), 1.56 (m, 2H, CH_2_), 1.43 (m, 2H, CH_2_); ^13^C NMR (125 MHz, CD_3_SOCD_3_):  *δ* = 170.1 CO, 133.4, 131.4 C=CH, 71.7, 66.8, 66.1 CH–OH, 31.5 CH(OH)CH_2_, 54.4, 51.6 NHCH_2_CH_2_NH_2_, 45.6, 39.3, 26.3, 24.6 CH_2_CH_2_CH_2_CH_2_; ESI-MS:  *m*/*z*: 585.99, 587.02, 588.00, 589.01, 589.98, 590.99, 592.01 [M + Cl]^−^.

*Compound* ***7d*** *(*PtL*^*d*^*Cl*_2_).* The procedure was similar to that for **7a** except that **6d** (L^d^) was used. Yield: 84%. Elemental analysis (%) calcd. for C_14_H_27_Cl_2_N_3_O_4_Pt·H_2_O: C 28.72, H 4.99, N 7.18; found C 28.65, H 4.78, N 7.25; ^1^H NMR (500 MHz, CD_3_SOCD_3_): *δ* = 7.85 (br s, 1H, H(a)), 6.95 (br s, 1H, H(c)), 6.42 (br s, 2H, H(f)), 6.23 (br s, 1H, H(2)), 4.84 (d, *J* = 3.5 Hz, 1H, 2-OH), 4.76 (d, *J* = 7.0 Hz, 1H, 3-OH), 4.59 (d, *J* = 3.5 Hz, 1H, 4-OH), 4.13 (m, 1H, H(2)), 3.76 (m, 1H, H(3)), 3.50 (m, 1H, H(4)), 3.04 (m, 2H, CH_2_), 2.82 (t, *J* = 5.5 Hz 2H, H(b), Pt satellites are observed as shoulders), 2.55 (m, 4H, H(d), and H(e), Pt satellites are observed as shoulders), 2.41 (m, 1H, H(5)), 1.91 (dd, *J* = 17.5 Hz, *J* = 4.5 Hz, 1H, H(5)), 1.53 (m, 2H, CH_2_), 1.38 (m, 2H, CH_2_), 1.20 (m, 2H, CH_2_); ^13^C NMR (125 MHz, CD_3_SOCD_3_): *δ* = 167.6 CO, 132.4, 132.2 C=CH, 71.5, 67.2, 66.1 CH–OH, 31.1 CH(OH)CH_2_, 54.3, 51.5 NHCH_2_CH_2_NH_2_, 45.8, 39.1, 29.2, 26.7, 24.3 CH_2_CH_2_CH_2_CH_2_CH_2_; ESI-MS:  *m*/*z*: 600.08, 601.03, 602.03, 603.05, 604.01 [M + Cl]^−^.

*Compound* ***7e*** *(*PtL*^*e*^*Cl*_2_).* The procedure was similar to that for **7a** except that **6e** (L^e^) was used. Yield: 80%. Elemental analysis (%) calcd. for C_15_H_29_Cl_2_N_3_O_4_Pt·1.5H_2_O: C 29.59, H 4.77, N6.90; found C 29.33, H 4.89, N 7.03; ^1^H NMR (500 MHz, CD_3_SOCD_3_): *δ* = 7.85 (br s, 1H, H(a)), 6.86 (br s, 1H, H(c)), 6.27 (br s, 2H, H(f)), 6.21 (br s, 1H, H(1)), 4.87 (d,  *J* = 3.5 Hz, 1H, 2-OH), 4.77 (d,  *J* = 6.5 Hz, 1H, 3-OH), 4.59 (*J* = 3.5 Hz, 1H, 4-OH), 4.18 (m, 1H, H(2)), 3.80 (m, 1H, H(3)), 3.57 (m, 1H, H(4)), 3.08 (t, *J* = 5.0 Hz, 2H, CH_2_), 2.85 (m, 2H, H(b), Pt satellites are observed as shoulders), 2.59 (m, 4H, H(d), and H(e), Pt satellites are observed as shoulders), 2.41 (m, 1H, H(5)), 1.96 (dd, *J* = 17.5 Hz, *J* = 4.5 Hz, 1H, H(5)), 1.56 (m, 2H, CH_2_), 1.42 (m, 2H, CH_2_), 1.26 (m, 4H, 2 × CH_2_); ^13^C NMR (125 MHz, CD_3_SOCD_3_) *δ* = 167.6 CO, 132.4, 132.2 C=CH, 71.4, 67.2, 66.0 CH–OH, 31.1 CH(OH)CH_2_, 54.3, 51.5 NHCH_2_CH_2_NH_2_, 45.8, 39.2, 29.4, 26.9, 26.6, 26.5 CH_2_CH_2_CH_2_CH_2_CH_2_CH_2_; ESI-MS:  *m*/*z*: 614.07, 615.07, 616.08, 617.08, 618.05, 619.03, 620.05 [M + Cl]^−^.

### 2.4. Cytotoxicity Assay *In Vitro*

The growth-inhibitory effects of selective synthetic compounds, K_2_PtCl_4_ and cisplatin on the BEL7404 human cancer cell lines, in a 72 h incubation, were measured by using the MTT method. The detailed procedure has been reported in our previous work [49].

### 2.5. Agarose Gel Electrophoretic Assay

DNA unwinding was determined by agarose gel electrophoretic assays through 1% (w/v) agarose gel with tris-acetate-EDTA (TAE) buffer, using pUC 19 plasmid DNA (0.5 *μ*L, the concentration was 20 ng/*μ*L) incubated with the ligands and compounds of various concentrations ranging from 20 to 260 *μ*M at 37°C in the dark for 3 h. Finally, the gels were stained with ethidium bromide (0.5 *μ*g/mL) for 30 min and visualized on a Bio-Rad gel imaging system.

### 2.6. ESI-MS Spectrometry

The reaction of five Pt(II) complexes with a model compound 5′-GMP was investigated using ESI mass spectrometry. The platinum complexes and disodium salt of 5′-GMP were mixed with a molar ratio of 1 : 2 in water at 37°C for 48 h. The mass range was  *m*/*z*  500–2000.

### 2.7. NMR Spectroscopy

The reaction of the Pt(II) complexes with excess (1 : 3) 5′-GMP was carried out in D_2_O within an NMR tube. The reaction mixtures were maintained at 37°C in the dark for 24 h, and then 1H NMR spectra data were recorded on a Bruker AV-500 NMR spectrometer.

### 2.8. Cell-Cycle Analysis

BEL7404 cell lines were maintained in Dulbecco's modified Eagle's medium with 10% fetal calf serum in 5% CO_2_ at 37°C. Cells were harvested by trypsinization and rinsed with PBS. After centrifugation, the pellet (105-106 cells) was suspended in 1 mL of PBS and kept on ice for 5 min. The cell suspension was then fixed by dropwise addition of 9 mL precooled (4°C) 100% ethanol under violent shaking. The mixed samples were kept at 4°C until use. For staining, cells were centrifuged, resuspended in PBS, digested with 150 mL RNAse A (250 *μ*g/mL), and treated with 150 mL P1 (100 *μ*g/mL) then incubated at 4°C for 30 min. PI-positive cells were counted with a FACScan fluorescence-activated cell sorter (FACS). The population of cells in each cell-cycle phase was determined using Cell Modi FIT software (Becton Dickinson).

## 3. Results and Discussion

### 3.1. Synthesis and Characterization

The ligands and platinum complexes were prepared following the previous reported methods [37–40, 42–48]. Prior to synthesizing the shikimic-carboxamide ligands 6a~e, side chains shown in Scheme 1 are required. 3-aminopropanol was N-protected with benzyloxycarbonyl groups, and the alcohol group of the resulting compound **(1)** was activated with methanesulfonyl chloride and reacted with excess 1,2-diaminoethane. The newly generated amine groups of the triamine derivative **2** were then protected with Boc groups, which produced the differentially protected triamine derivative **3**. Selective deblocking the N-Cbz group gave the unstable amine **4**, which then reacted selectively with shikimic acid to produce shimiccarboxamide **5**. This was converted to shikimic carboxamide ligand **6** by deblocking the N-Boc group, the precursor ligand for the desired platinum complexes **(7)**. The ligands **6a~e** (L^a–e^) reacted easily with K_2_PtCl_4_ in the dark at room temperature, and gave the corresponding platinum complexes **7a~e** (PtL^a–e^Cl_2_). Complexes PtL^a–e^Cl_2_ are soluble in water, with solubilities of 40.3, 32.9, 21.7, 17.0, and 12.6 mg/mL at 298 K, respectively. These compounds were characterized by elemental analysis, ^1^H NMR, ^13^C NMR, and ESI-MS spectroscopies.

All ligands comprise three potential nitrogen donor sites (two amines and one amide). Hence, upon reaction with K_2_PtCl_4_, the ligands could bind to the metal center in a bidentate manner (two possible isomers: 2 × amine, 1 × amine, and 1 × aimde), or even in a tridentate way. Although the results of elemental analysis of the platinum(II) complexes are supportive of a bidentate binding, it does not indicate which isomer is formed, or whether both are formed. In order to confirm the isomer formed, the capillary electrophoresis, LC-MS, and detailed comparison of ^1^H, ^13^C NMR were carried out. As shown in Figure S1, supplementary material available online at http://dx.doi.org/10.1155/2013/565032 (see ESI†), only a single peak in their capillary electrophoresis plots was observed for PtL^b^Cl_2_, PtL^c^Cl_2_, PtL^d^Cl_2_, and PtL^e^Cl_2_ complexes, respectively, which indicated that the product (isomer) was pure and unique; furthermore, the purity of the product (isomer) was confirmed by the results of LC-MS spectra analysis (see Figure S2, ESI†). To determine the ligand coordinate mode in the product (isomer), we in detail investigated the NMR difference between the Pt(II) complexes and free ligands. As shown in Table 1, the differences of  Δ*δ*(H^a^)  between L^a–e^ ligands and PtL^a–e^Cl_2_ complexes are less; however, those of Δ*δ*(H^c^) and Δ*δ*(H^f^) are very large; accompanying this trend, those of Δ*δ*(H^b^); Δ*δ*(H^d^), and Δ*δ*(H^e^) are moderate. The large proton chemical shift differences of two amines mainly induced by the bidentate coordinate mode of two amines of the ligands. Due to the coupling interaction, the adjacent proton chemical shifts also generated moderate changes. These observations were further confirmed by ^13^C NMR shift differences (Δ*δ*) between L^a–e^ ligands and PtL^a–e^Cl_2_ complexes. As shown in Table 2, the differences of Δ*δ*(C^a^) and Δ*δ*(C^b^) between L^a–e^ ligands and PtL^a–e^Cl_2_ complexes are very less, however, those of  Δ*δ*(C^c^); Δ*δ*(H^d^), and Δ*δ*(C^e^) are very large, which could be resulted from the bidentate coordinate mode of two amines of the ligands. It should be pointed out that platinum satellites were observed as shoulders in the ^1^H NMR spectra of PtL^a–e^Cl_2_ complexes [50–52].

Based on the above mentioned, we could conclude that only one isomer of PtL^a–e^Cl_2_ formed through the bidentate coordinate mode of two amines under the present experimental conditions and their chemical structures were shown Scheme 1.

### 3.2. Cytotoxic Activity *In Vitro*

In an *in vitro* assay, the platinum complexes with diamine coupled shikimic acid ligands are weakly active against BEL7404 cancer cell lines (see Table 3, the data for compound **7a** (PtL^a^Cl_2_) is not available due to its low yield) but do not exhibit activity against SGC-7901, SK-OV-3, CNE-2, and HeLa cancer cell lines under the tested concentrations. Therefore, the platinum complexes with diamine coupled shikimic acid ligands display a certain extent selective cytotoxicity. And their corresponding ligands do not exhibit activity. Their activities depend on the carbon linker length, in which the IC_50_ values increase at longer carbon linker. Though these platinum(II) complexes with shikimic acid-based ligands possess high water solubility, they are less active than cisplatin, Pt-shikimato complexes [41], carbohydrate-metal complexes [32], and carbohydrate-linked cisplatin analogues [20]. Such observations should be correlated their low lipophilicity. Recently, lipophilicity has been considered a crucial aspect for the cytotoxicity of platinum complexes [51–56]. The platinum antitumour agents must enter cells before reaching their main biological target, namely, DNA. Their distribution within the body and, hence, their activity are to a large extent determined by their lipophilicity [57]. It is believed that the more lipophilic a complex, the higher its cytotoxicity [58]. Since PtL^b–e^Cl_2_ complexes attached a high hydrophilic shikimato group which resulted in a low lipophilicity of the whole PtL^b–e^Cl_2_ complexes. Thus, it is expected that these platinum complexes do not effectively enter the cells and lead to a low activity [51–58]. 

### 3.3. DNA Unwinding and Cleavage Studies

Since DNA is the primary target of Pt(II)-based antitumor complexes, the DNA binding behaviors of the PtL^a–d^Cl_2_ complexes have been studied via agarose gel electrophoresis. 

**Figure 1:**
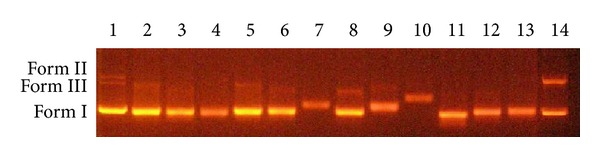
Electrophoretic mobility of pUC19 plasmid DNA. Lane 1: DNA, Lane 2~4: DNA + PtL^a^Cl_2_ (50, 100, 200 *μ*M), Lane 5~7: DNA + PtL^b^Cl_2_ (50, 100, 200 *μ*M), Lane 8~10: DNA + PtL^c^Cl_2_ (50, 100, 200 *μ*M), and Lane 11~13: DNA + PtL^d^Cl_2_ (50, 100, 200 *μ*M), and Lane 14: DNA + CDDP (40 *μ*M).

Figure 1 and Figure S1 show the results of agarose gel electrophoresis on pUC19 plasmid DNA, in which three forms are present: supercoiled DNA (Form I) as the dominant components, nicked or open circular DNA (Form II), and linear DNA (Form III) bound with ligands and homologous Pt(II) complexes. The complexation of ligands L^a–d^ did not produce any change in the migration rate during agarose gel electrophoresis (L^c^ as the example, Figure S3, ESI†). 

Due to these platinum(II) complexes having similar structure, herein, we just selected four of them as representatives. As shown in Figure 1, though the presence of PtL^a^Cl_2_ did not reduce the electrophoretic mobility of supercoiled DNA obviously, the proportion of supercoiled form deccreased obviously upon increasing the concentrations of 100 and 200 *μ*M. While the presence of PtL^b^Cl_2_, PtL^c^Cl_2_, and PtL^d^Cl_2_, especially PtL^c^Cl_2_, has induced significant reduction of the electrophoretic mobility of supercoiled DNA, all of them had concentration dependence. It seems that all platinum(II) complexes exhibit high binding affinity to DNA, and covalent binding mode of these complexes to DNA is proposed.

### 3.4. The Interaction with 5′-GMP

As guanine-N7 is the preferable binding site in DNA to bind with platinum-based drugs [59], we investigated the reaction of complexes PtL^b^Cl_2_, PtL^c^Cl_2_, and PtL^d^Cl_2_ with 5′-GMP using ESI-MS and 1H NMR spectrometry.

#### 3.4.1. ESI-MS Analysis

**Figure 2:**
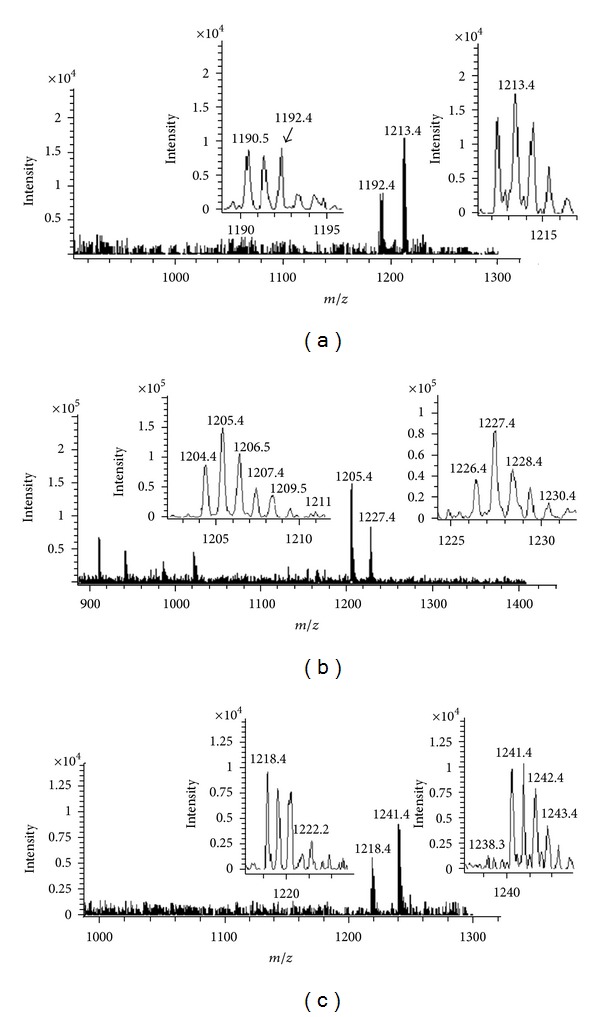
The ESI-MS spectra showing the formation of 5′-GMP adducts with complexes PtL^b^Cl_2_ (a), PtL^c^Cl_2_ (b), and PtL^d^Cl_2_ (c).

Two peaks  *m*/*z*  1213.4 and 1192.4 in Figure 2(a) are assigned to one negatively charged species [PtL^b^(5′-GMP)^2^-4H + Na]^−^ (C_32_H_47_N_13_NaO_20_P_2_Pt, calcd. 1213.8) and [PtL^b^(5′-GMP)_2_-3H]^−^ (C_32_H_48_N_13_O_20_P_2_Pt, calcd. 1191.8), respectively. Peaks  *m*/*z*  1227.4 and 1205.4 in Figure 2(b) are assigned to Pt-DNA adducts [PtL^c^(5′-GMP)_2_-4H + Na]^−^ (C_33_H_49_N_13_NaO_20_P_2_Pt, calcd. 1227.8) and [PtL^c^(5′-GMP)_2_-3H]^−^ (C_33_H_50_N_13_O_20_P_2_Pt, calcd. 1205.9), respectively.  Figure 2(c) shows peaks  *m*/*z*  1241.4 and 1218.4, which are attributed to the species [PtL^d^(5′GMP)_2_-4H + Na]^−^ (C_34_H_51_N_13_NaO_20_P_2_Pt, calcd. 1241.9) and [PtL^d^(5′-GMP)_2_-3H]^−^ (C_34_H_52_N_13_O_20_P_2_Pt, calcd. 1219.9), respectively. These results suggested that the three complexes reacted with 5′-GMP by covalent bonding, and two chloro ligands were removed.

#### 3.4.2. ^1^H NMR Analysis

We took the reaction of compounds **7c** (PtL^c^Cl_2_) with 5′-GMP as an example to confirm the coordinate site of 5′-GMP using ^1^H NMR spectroscopy. In all cases, the typical characters H8 of 5′-GMP downshift (8.1 ppm for H8 of free 5′-GMP) and the appearance of H8 signals corresponds to platinum adducts (8.5 ppm for H8 of bis-bound and 8.6–8.8 ppm for H8 of monobound 5′-GMP) [60–62]. Dijt et al. reported that the N7 position indicated the absence of a protonation effect at low pH in the pH-dependent behavior of H8 from the end products [63].

**Figure 3:**
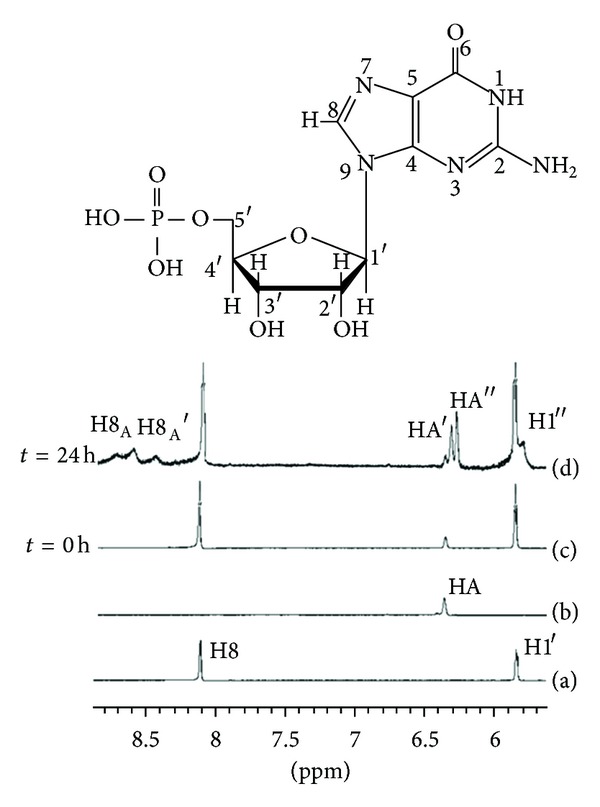
Selected ^1^H NMR spectra for GMP (a), complex PtL^c^Cl_2_ (b), the reaction of complex PtL^c^Cl_2_ with 3 equiv. of 5′-GMP in D_2_O after being incubated at 37°C for 0 h (c) and 24 h (d), respectively.

In contrast to the individual component, the ^1^H NMR spectroscopy of the reaction mixture of 5′-GMP and PtL^c^Cl_2_ showed clear changes after an incubation of 24 h at 37°C (Figure 3). The assignments for the representative peaks have been listed in Table 4. Peaks at 8.65 ppm for H8_A_ and at 8.49 ppm for H8_A_′ were observed at 24 h and downfield shifted from the H8 signal at 8.11 ppm, which indicates the formation of Pt-GMP mono-bound and bis-bound adducts. Meanwhile, the signal of sugar H1′ at 5.83 ppm partially shifted to 5.78 ppm, and new signals at 6.31 ppm and 6.27 ppm assigned to PtL^c^Cl_2_ appeared after incubation with 5′-GMP. These changes suggest that both sugar H1′ and alkene proton were shield due to the platination of N7 of 5′-GMP. The upfield shift of sugar H1′ resonance has been observed in the ^1^H NMR spectrum of *cis*-[Pt(GMP)_2_(NH_3_)_2_]^2−^ [64–66]. The results show that PtL^c^Cl_2_ can bind to 5′-GMP upon the platination of N7 of guanine, consistent with the ESI-MS results mentioned above.

In summary, these water-soluble platinum(II) complexes can bind to DNA and 5′-GMP, and the low cytotoxicity should correlate their low lipophilicity, but the cellular uptake and detailed action mechanism need further investigation in the continuing work.

### 3.5. S-Phase Cell-Cycle Arrest

To determine whether cellular DNA is a major target of the water-soluble Pt(II) complexes, we studied the cell-cycle profiles of PtL^b^Cl_2_ and PtL^c^Cl_2_ treated cancer cells (because compounds **7a~e** have similar structures; here, only select PtL^b^Cl_2_ and PtL^c^Cl_2_ to investigate their cell-cycle profiles). Cell-cycle analysis was performed, and flow cytometry was used to assess the DNA content of cells stained with propidium iodine, which enables us to quantify the total cellular populations in different phases of the cell cycle (G_0_/G_1_, S, and G_2_/M). The flow-cytometric data for the BEL7404 cells treated with PtL^b^Cl_2_ and PtL^c^Cl_2_ were presented in Table 5. Upon treating cells with PtL^b^Cl_2_ and PtL^c^Cl_2_ (250 *μ*M) for 72 h, the cell-cycle arrest has enhanced at G_2_ phase, resulting in concomitant increases in the G_2_ phase population and decrease in the S phase population. The direct interaction of PtL^b^Cl_2_ and PtL^c^Cl_2_ with DNA has been examined by agarose gel electrophoresis. It was found that PtL^b^Cl_2_ and PtL^c^Cl_2_ are able to alter DNA configuration. Given all these results, DNA may be a crucial cellular target of PtL^b^Cl_2_ and PtL^c^Cl_2_ in inducing its cytotoxicity.

## 4. Conclusion Remarks

We have synthesized five water-soluble platinum(II) complexes: PtL^a–e^Cl_2_ (L^a–e^ = SA-NH(CH_2_)_*n*_NHCH_2_CH_2_NH_2_, *n* = 2–6) through the reactions of Pt(II) with TCM active ingredient, shikimic acid, and coupled aliphatic amine with different carbon linkers (NH_2_(CH_2_)_*n*_NHCH_2_CH_2_NH_2_, *n* = 2–6). These Pt(II) complexes interact with DNA by covalent binding, which blocks the DNA synthesis and replication thus inducing low cytotoxicity against cells like BEL7404. The low cytotoxicity should correlate with their low lipophilicity. In addition, PtL^b^Cl_2_, PtL^c^Cl_2_, and PtL^d^Cl_2_ could react with 5′-GMP to form monoGMP and bisGMP adducts via hydrolysis. The cell-cycle analysis revealed that PtL^b^Cl_2_ and PtL^c^Cl_2_ cause G_2_-phase cell arrest.

## Supplementary Material

Figure S1: Capillary electrophoresis plot of four platinum(II) complexes. The electrophoresis experimental conditions: running buffer pH 7.0, 5 mmol/L PBS buffer (containing 10% methanol); capillary column 30 cm×50 *µ*m i.d.; detected wavelength *λ* = 214 nm; applied voltage 20 kV; input sample time 8 s; concentration 1×10^−3^ mol/L. Figure S2: LC-MS spectra of for platinum(II) complexes. Analytical separations were carried out on a reversed phase column (XB-C18, 3*μ*m, 2.1×150mm,) with detection at 300 nm. Mobile phase A: water, B: methanol, the flow rate was 0.3 mL min^−1^, The gradient (Solvent B) was as follows: 0% to 2% within 3 min, 2% to 5% from 3 to 5 min, reset to 10% from 5 to 30 min. Figure S3: Electrophoretic mobility of pUC19 plasmId DNA. DNA was equilibrated with increasing concentrations of L^c^ for 3 h at 37. Lane 1: DNA, Lane 2~6: DNA + L3 (10, 50, 100, 200, 300*µ*M).

## Figures and Tables

**Table 1:** ^
1^H NMR shifts difference (Δ*δ*, ppm) between L^a–e^ ligands and PtL^a–e^Cl_2_ complexes.

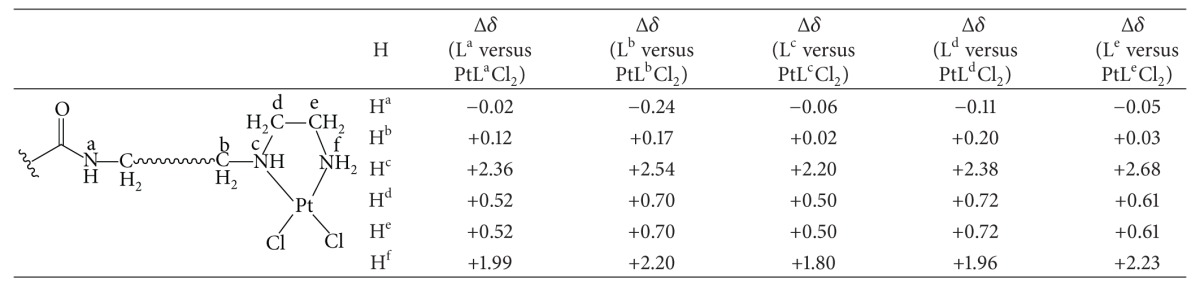

**Table 2:** ^
13^C NMR shifts difference (Δ*δ*, ppm) between L^a–e^ ligands and PtL^a–e^Cl_2_ complexes.

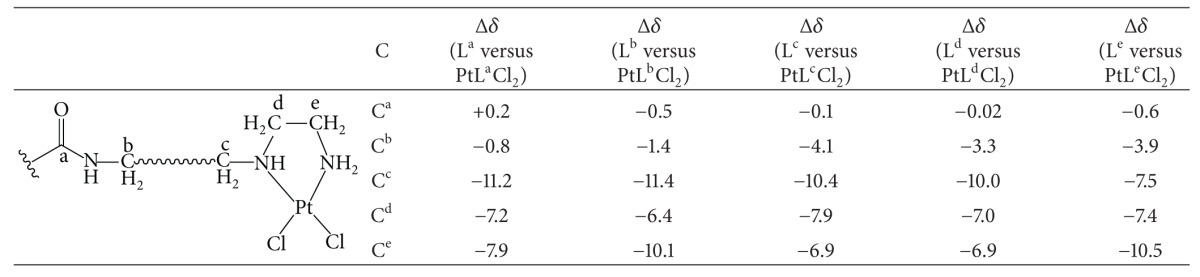

**Table 3:** IC_50_ values (*μ*M) for the water-soluble platinum(II) complexes, cisplatin in BEL7404 cancer cell lines^a^.

PtL^b^Cl_2_	PtL^c^Cl_2_	PtL^d^Cl_2_	PtL^c^Cl_2_	Cisplatin
289.3 ± 18.6	298.4 ± 22.5	387.2 ± 7.8	391.7 ± 10.6	98.0 ± 17.4

^a^IC_50_ values are presented as the mean ± SD (standard error of the mean) from five separated experiments. Cisplatin was used as positive control.

**Table 4:** Assignments of the selected peaks in ^1^H NMR spectra for reaction of complex PtL^b^Cl_2_ with 5′-GMP.

Peak	*δ* (ppm)	Assignment
H8	8.11	H8, free 5′-GMP
H1′	5.83	H1′, ribose in free 5′-GMP
HA	6.36	HA, in free Pt(L4)Cl_2_
H8_A_	8.65	H8, mono-GMP adduct
H8_A_′	8.49	H8, bis-GMP adduct
H1′′	5.78	H1′, ribose in GMP adduct
HA′	6.31	HA, mono-GMP adduct
HA′′	6.27	HA, bis-GMP adduct

**Table 5:** Induction of cell-cycle arrest in BEL7404 cells after treatment with PtL^b^Cl_2_, PtL^c^Cl_2_.

	Dip G_1_ (%)	Dip G_2_ (%)	Dip S (%)
Pt(L^b^)Cl_2_	49.26	27.09	23.64
Pt(L^c^)Cl_2_	50.57	21.21	28.22
Untreated	49.92	14.74	35.34

## Abbreviations

BEL7404: Liver cancer cell
BSA: Bovine serum albumin
CDDP: Cisplatin
CNE-2: Human nasopharyngeal carcinoma cells
ESI-MS: Electrospray ionization mass spectrum
5′-GMP: 5′-guanosine monophosphate
HeLa: Human cervical cancer cells
MTT: 3-[4,5-dimentylthiazole-2-yl]-2,5-diphenpyltetra-zolium bromide
PBS: Phosphate-buffered saline
SGC-7901: Human gastric adenocarcinoma cells
SK-OV-3: Human ovarian cancer cells
TBE: Tris, boric acid, EDTA.
