# Supplementary material for: Synthesis, Characterization, and Interaction with Biomolecules of Platinum(II) Complexes with Shikimic Acid-Based Ligands
Source: Bioinorg Chem Appl. 2013 Mar 5;2013:565032. doi: 10.1155/2013/565032 (PMC3603162; doi:10.1155/2013/565032)
Supplement: Supplementary file 1 — Figure S1: Capillary electrophoresis plot of four platinum(II) complexes. The electrophoresis experimental conditions: running buffer pH 7.0, 5 mmol/L PBS buffer (containing 10% methanol); capillary column 30 cm×50 µm i.d.; detected wavelength λ = 214 nm; applied voltage 20 kV; input sample time 8 s; concentration 1×10^−3^ mol/L. Figure S2: LC-MS spectra of for platinum(II) complexes. Analytical separations were carried out on a reversed phase column (XB-C18, 3μm, 2.1×150mm,) with detection at 300 nm. Mobile phase A: water, B: methanol, the flow rate was 0.3 mL min−1, The gradient (Solvent B) was as follows: 0% to 2% within 3 min, 2% to 5% from 3 to 5 min, reset to 10% from 5 to 30 min. Figure S3: Electrophoretic mobility of pUC19 plasmId DNA. DNA was equilibrated with increasing concentrations of Lc for 3 h at 37. Lane 1: DNA, Lane 2~6: DNA + L3 (10, 50, 100, 200, 300µM). [file 565032.f1.pdf]

Electronic Supplementary Information (ESI)

Synthesis, characterization and interaction with biomolecules of platinum(II) complexes with shikimic acid-based ligands

Yan Peng, Min-Min Zhang, Zhen-Feng Chen,* Kun Hu, Yan-Cheng Liu, Xia Chen and Hong Liang*

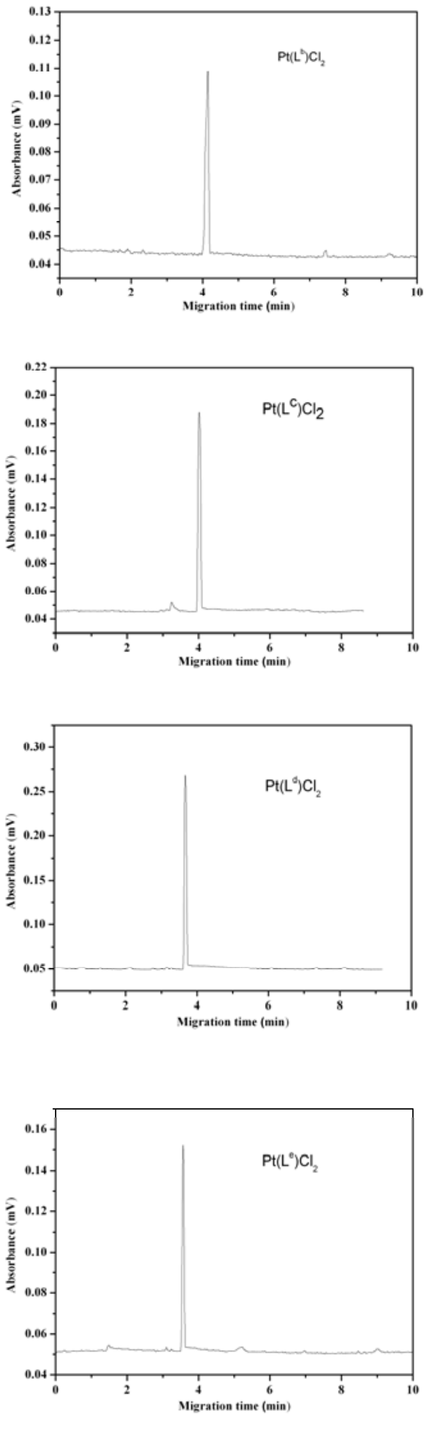

Figure S1. Capillary electrophoresis plot of four platinum(II) complexes. The electrophoresis experimental conditions: running buffer pH 7.0, 5 mmol/L PBS buffer (containing 10%

methanol); capillary column 30 cm×50 μm i.d.; detected wavelength λ = 214 nm; applied voltage 20 kV; input sample time 8 s; concentration 1×10^{-3} mol/L.

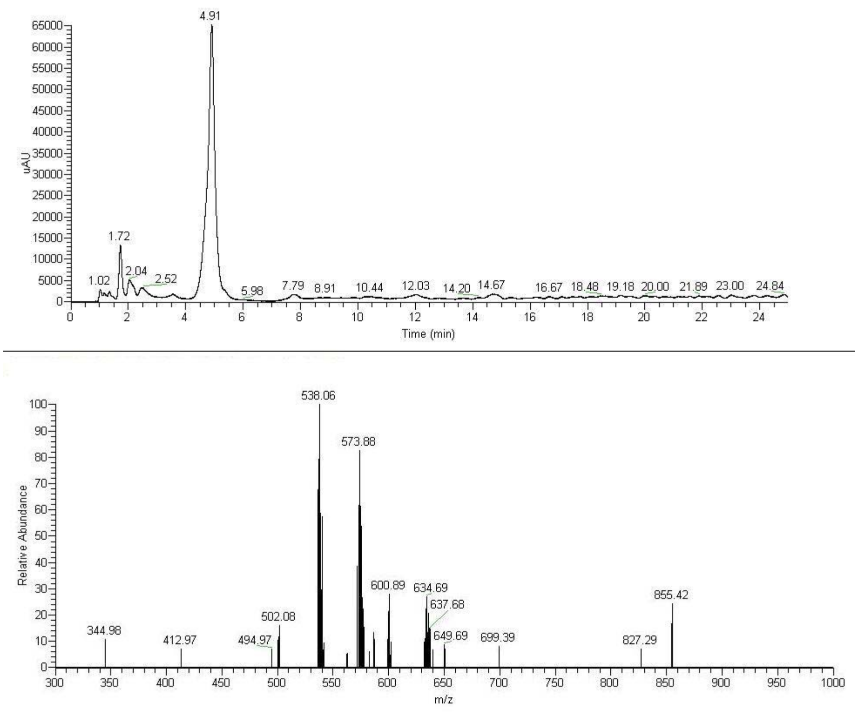

LC-MS spectra for PtL^bCl_2

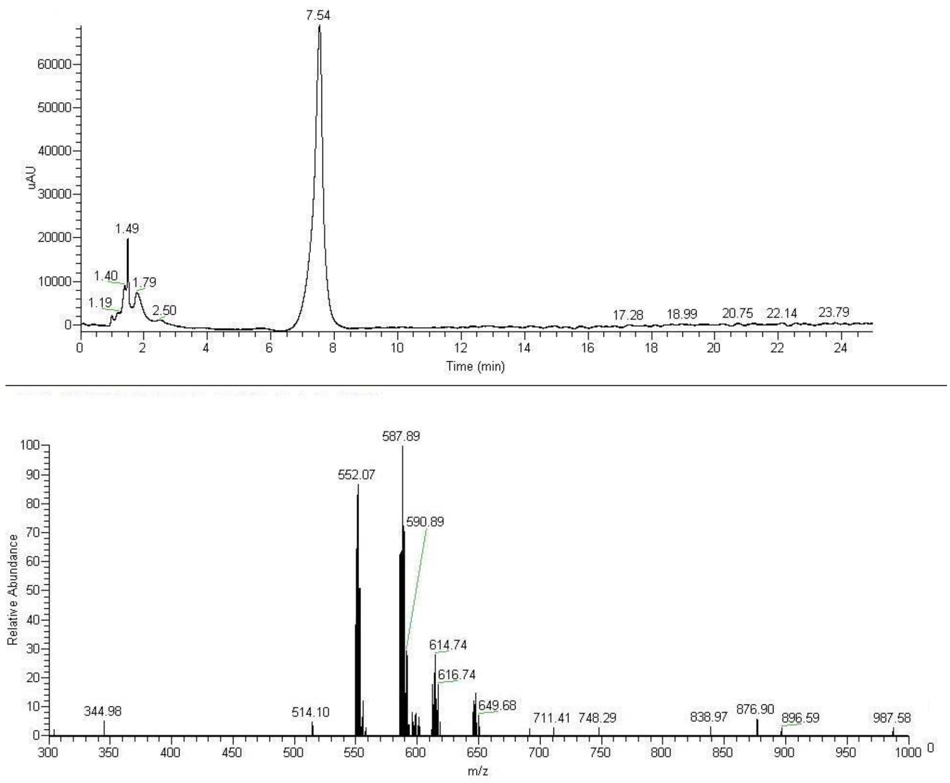

LC-MS spectra for PtL^cCl_2

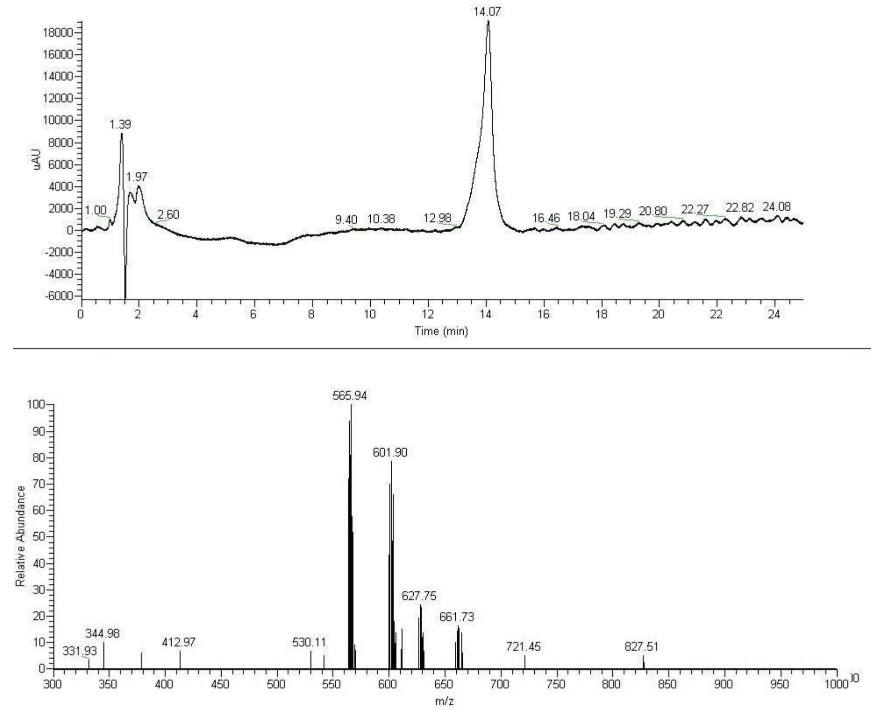

LC-MS spectra for PtL^dCl_2

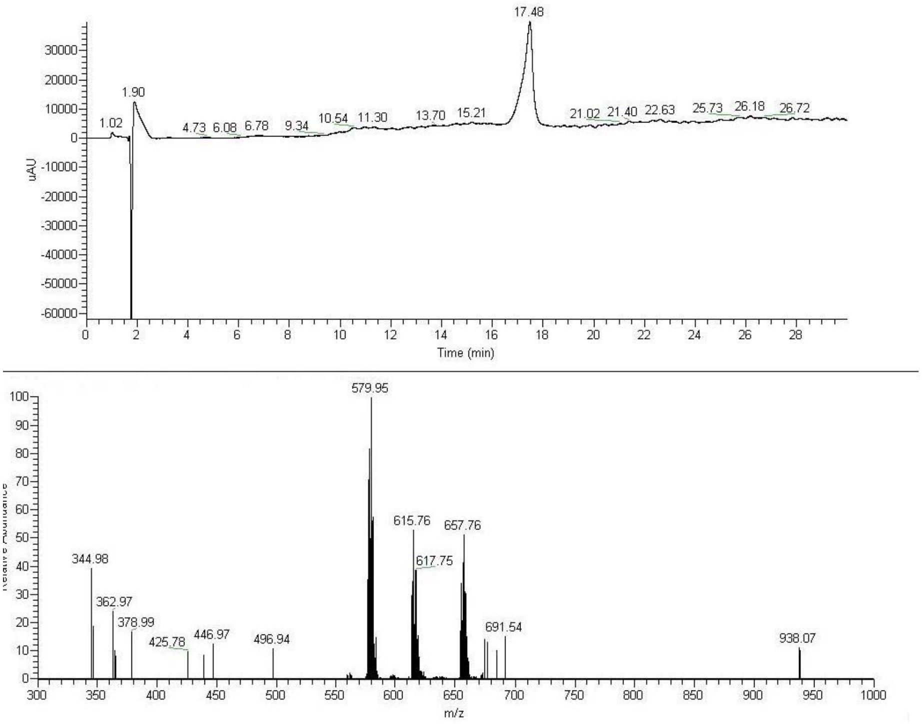

LC-MS spectra for PtL^eCl_2

Figure S2. LC-MS spectra of for platinum(II) complexes. Analytical separations were carried out on a reversed phase column (XB-C18, 3μm, 2.1×150mm,) with detection at 300 nm. Mobile phase A: water, B: methanol, the flow rate was 0.3 mL min^{-1}, The gradient

(Solvent B) was as follows: 0% to 2% within 3 min, 2% to 5% from 3 to 5 min, reset to 10% from 5 to 30 min.

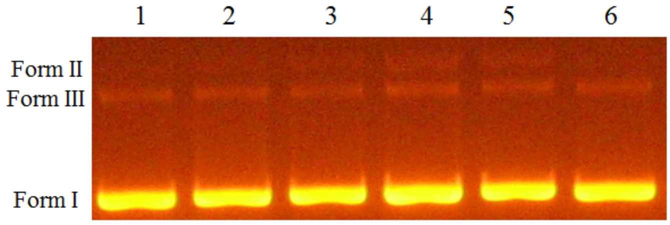

Figure S3. Electrophoretic mobility of pUC19 plasmId DNA. DNA was equilibrated with increasing concentrations of L^c for 3 h at 37℃. Lane 1: DNA, Lane 2~6: DNA + L3 (10, 50, 100, 200, 300μM).
